# Experiences of undergraduate nursing students during clinical practice at health facilities in Western Cape, South Africa

**DOI:** 10.4102/curationis.v44i1.2127

**Published:** 2021-04-08

**Authors:** Fundiswa P. Fadana, Hilda F. Vember

**Affiliations:** 1 Department of Nursing, Faculty of Health and Wellness Sciences, Cape Peninsula University of Technology, Cape Town, South Africa

**Keywords:** clinical environment, clinical practice, experiential learning, undergraduate nursing students, clinical competence

## Abstract

**Background:**

This study explored the experiences of undergraduate nursing students during clinical practice at healthcare facilities in the Boland Overberg area in Western Cape, South Africa. Few studies have been done on experiences of nursing students during clinical practice. However, there are still inadequacies, which lead to the deterioration of clinical practice quality.

**Objectives:**

To explore and describe the experiences of undergraduate student nurses during clinical practice in healthcare facilities in Boland Overberg, in Western Cape, South Africa.

**Method:**

A qualitative, exploratory descriptive design was applied. Data collection was done using focus-group interviews to ascertain the undergraduate student nurses’ experiences during clinical practice in healthcare facilities in the Boland Overberg area in Western Cape Region. Thirty-eight undergraduate nursing students from Boland Campus were selected, using purposive sampling. The sample size was based on data saturation. Colaizzi’s method of coding and thematic content analysis was used to interpret the data. Ethical principles were adhered to.

**Results:**

After data analysis, the following themes emerged: clinical learning environment, challenges and inability to reach objectives.

**Conclusion:**

During clinical practice in healthcare facilities, students were confronted with dilemmas which must be addressed with proper planning to decrease the challenges in clinical education of future nurses. The findings can be used in planning of nursing education, could provide help to develop effective clinical teaching strategies in nursing education and to support these undergraduate nursing students.

## Introduction

Nursing is a practice-based discipline. Hence, clinical practice is regarded as an important component of nursing education (Arpanantikul & Pratoomwan 2017:121; Kalyani et al. 2019:1). However, healthcare settings are not always the perfect environment for learning (Mamaghani et al. 2018:216). During clinical practice, various situations can arise, hindering the learning performances of students (Kalyani et al. 2019:1). Clinical practice, together with the student’s experience assisted the students to acquire the necessary skills and knowledge to become capable of supporting human lives through the practice of nursing (Mueller, Naragon & Smith 2016:3). Mueller et al. (2016:3) stated that if essential learning opportunities are missed, or the students are exposed to negative experiences, these nursing students may not be able to grow in learning or they will remain incompetent in the field. This may lead to patient safety issues. According to Bazrafkan and Kalyani (2018:4), the quality of nursing care depends on the quality of clinical education provided to the student nurses. The better the quality of clinical education provided to the student nurse, the more successful and professional students will graduate.

In South Africa, different nursing programmes are offered. One of these programmes is the Bachelor of Technology in Nursing (BTech Nursing) which is an undergraduate program, governed by Government Notice No. R.425 0f 22, February 1985. The South African Nursing Council is a regulatory body that guides nursing training in South Africa (SANC). According to SANC (1985:21), B.Tech nursing students are expected to complete 4000 clinical hours over four years. For students to fulfil this requirement, they are placed in healthcare facilities where they practice clinical nursing.

In the researcher’s own experience as a nurse lecturer, when the undergraduate nurses return from their placement areas, they usually reflect on their experiences whilst they were doing clinical practice in healthcare facilities. Students mostly complained about factors at healthcare facilities that are challenging and not conducive to learning. According to Jamshidi et al. (2016:1), clinical practice is influenced by the environment where it takes place.

It has been proved that even though the clinical practice area is important, it is also challenging to the students learning (Mamaghani et al. 2018:222). Experiences in healthcare areas (real world) may be positive or negative (Arpanantikul & Pratoomwan 2017:122). The literature identified a few common factors experienced by undergraduate nursing students during clinical practice. These factors were either related to the unfriendly clinical environment, shortage of staff and theory-practice gap (Kalyani et al. 2019:5; Zenani 2016:VI).

As a nurse lecturer, the researcher found that the students were not satisfied with their clinical learning during placement and complained about the environment not being conducive to learning. These complaints reflected in their poor performance during the objective structured clinical examination (OSCE). During the OSCEs, students are assessed on their clinical skill competencies. It was during these OSCEs that the researcher noticed that students were neither confident nor clinically skilled and competent, performing poorly. The complaints of the students, and the students’ performance during OSCE prompted an interest in formally investigating the experiences of the undergraduate nurses during clinical practice in healthcare facilities. Despite all studies done on the clinical practice of an undergraduate student nurse, clinical practice in healthcare facilities ‘in real situations’, is still a problem (Atakro et al. 2019:1).

In South Africa, there are a few studies that were done pertaining to the experiences of undergraduate nursing students during experiential learning. However, there are still inadequacies, which lead to the deterioration of clinical practice quality (Mabuda, Potgieter & Alberts 2008; Matshotyana, Van Rooyen & Du Randt 2015; Mothobi 2017). Through this project, the researcher hoped to get a better understanding of student nurses’ experiences whilst doing experiential learning in healthcare facilities in the Boland Overberg area in the Western Cape, South Africa. The knowledge and understanding of how these undergraduate students experience things would help the nurse educators as well as the clinical staff in clinical settings to develop appropriate strategies to facilitate the progress of clinical competence of student nurses and to improve the quality of nursing education.

The research question was, ‘What are the experiences of undergraduate nursing students during their experiential learning in public healthcare facilities in the Boland Overberg area of the Western Cape?’

## Design and method

A qualitative, exploratory descriptive and contextual design was used to explore and understand the experiences of undergraduate nursing students during clinical practice. Qualitative methods study human experiences from the perspective of the participants, within the context in which the phenomenon takes place (Brink, Van der Walt & Van Rensburg 2018:105; Gray, Grove & Sutherland 2017:62; Grove, Gray & Burns 2015:67; Polit & Beck 2017:471). A qualitative method was chosen because it elicits rich and in-depth findings that provide a unique appreciation of the reality of different experiences (Polit & Beck 2017:471). Qualitative method allowed the researcher to explore the experiences of the undergraduate student nurses during clinical practice, and the meaning these experiences hold for these students.

The researcher also chose a qualitative method as the focus was on practices, behaviour, and attitudes, all of which are part of the lived experiences of student nurses (Polit & Beck 2017:59). This design was followed to explore and describe the experiences of second, third and fourth-year undergraduate nursing students in real-time, and to describe and understand what was happening during the clinical practice of undergraduate nursing students in real situations (Grove et al. 2015:77). This design helped the researcher to collect more information about the experiences of undergraduate nurses during their clinical learning in healthcare facilities and to identify challenges these students faced during their clinical practice in placement areas (Brink et al. 2018:96). This research took place at a rural university campus in Western Cape.

### Population and sample

The entire population of this study included 184 nursing students registered for the B.Tech programme at this rural campus in Western Cape. Purposive sampling was used. Thirty-eight participants, between the ages of 19–34 years were selected deliberately based on the clinical knowledge they had, as they had been exposed to clinical practice in the healthcare facilities (Gray et al. 2017:345). The sample size in this study was determined by the depth and richness of information required to gain insight into a phenomenon and understand it (Polit & Beck 2017:397). The researcher continued with interviews until data saturation (Grove et al. 2015:274). This study included all second to the fourth year undergraduate students registered in this rural campus for the B.Tech nursing course; the students were those who had not failed during her/his years of study and had to be 18 years of age and older. Participants could be female or male. First-year students were excluded since at the time of data collection, they had not yet been exposed to clinical practice.

### Data collection

Focus-group interviews were used as a mode of data collection to understand the experiences of undergraduate nurses during clinical practice in healthcare facilities. Focus groups interviews were conducted between 25 November 2016 and 16 March 2017. Interviews were conducted in English. The first two focus groups were conducted on 25 November 2016. Data collection was interrupted by #FeesMustFall protest prevalent on all university campuses nationally. The rest of the data-collection process was from 13 February 2017 to 16 March 2017. Each interview lasted approximately 45–60 min. Interviews were organised around a set of open-ended questions.

Interviews were conducted in one of the study halls at this campus. This was the choice of the participants. Eight focus groups were made, of which six of these groups consisted of five participants each. The other two groups comprised of four participants only as some students did not arrive for the interviews. According to Gray et al. (2017:263), each focus group should consist of four to twelve participants.

These focus groups consisted of female and male students from the second, third and fourth-year levels of study. In this study, focus groups were used to understand the experiences of undergraduate nurses during clinical practice in healthcare facilities. An Olympus digital voice recorder was utilised to do all the recordings. Each interview lasted approximately 45–60 min.

### Data analysis

Data analysis was done following Colaizzi’s (1978) seven-step method of qualitative data analysis discussed in Polit and Beck (2017:540). Recorded data from the focus groups were transcribed word for word by the researcher herself. This allowed the researcher to engage intensely with the data. This was done by listening to the focus group recordings more than once to maintain the integrity of the typed transcriptions (Grove et al. 2015:88). The transcripts were read repeatedly until the researcher was familiar with the data. The supervisor assisted with the coding and the formulation of themes to avoid any bias. The researcher used the keywords and typed the relevant data on the left, two-thirds of the page, whilst the right margin was used to write codes (Saldaña 2013:16). The coding process assisted the researcher in organising and grouping similar coded data into themes (Gray et al. 2017:271).

### Trustworthiness of the study

Throughout the study, the researcher adhered to the four basic frameworks to ensure rigour. Regarding the trustworthiness of the data collected, credibility was used to judge the accuracy and correctness of the research findings (Polit & Beck 2017:559). Member checks and peer debriefing was used to achieve the credibility of this study (Brink et al. 2018:111). After the transcriptions were done, the researcher made use of member checks to allow members to verify that the transcriptions were a true reflection of what they had said during the focus group interviews (Polit & Beck 2017:564).

In this study, peer checking was done by the researcher’s colleagues, and the researcher kept a journal of reflection in order to cross-reference and minimise personal bias during data analysis (Anney 2014:279). The supervisor also assisted with the coding of the data to reduce risk of being biased (Polit & Beck 2017:562). Both, supervisor and the author had the same scripts to develop the coding, and matching codes were identified.

Regarding confirmability, the researcher used the audit trail technique to check whether conclusions and interpretations could be traced to the source. All raw data were audiotaped with the permission of participants and then transcribed verbatim before analysis was done. The supervisor also assisted with the coding of the data (Brink et al. 2018:181), after which various themes were identified (Polit & Beck 2017:59). The transcripts and audio recordings will be kept safe for five years in a locked safe at the university, to which only the supervisor will have access, so that these records can be used to cross-check results whenever required.

### Ethical considerations

Approval to conduct the study was received from the Cape Peninsula University of Technology Faculty of Health and Wellness Sciences Research Ethics Committee: NHREC: REC- 230408-014.

The researcher started collecting data as soon as approval from the Ethics Committee of the university was granted. Permission and support were also granted by the head of the College. The researcher complied with the following ethical principles as out lined by Polit and Beck (2017:139): principles of autonomy, beneficence and non-beneficence, justice, respect for the people involved and maintaining of confidentiality to protect the participants, were upheld throughout the research process. All participants were informed of their right to withdraw from the study at any time if they felt like doing so. Informed consent was received from participants before the commencement of data. The researcher invited the participants to the study by writing to them individually. The invitation letter informed the participants of the purpose of the study, the voluntary nature of participation, what was expected of participants, how data would be collected and the duration of the interviews. The participants signed a written consent form after the researcher had explained the details again, after they had read the invitation letter. The participants were assured of anonymity to protect them from any harm, throughout the interview process.

## Results

The results of this study revealed that undergraduate students were faced with challenging experiences. After coding, experiences of undergraduate nursing students were categorised. One theme and nine sub-themes were identified (see Table 1):

**TABLE 1:** Themes and subthemes.

Theme	Subthemes
**Challenges**	Unhealthy interpersonal relationships
	Poor communication
	Integration of theory and practice
	Anxiety
	Students were neglected
	Violence
	Discrimination
	Inability to reach objectives
	Congestion of students in a clinical setting

## Theme: Challenges

This main theme consists of seven sub-themes which are poor interpersonal relationships, poor communication skills, theory-practice gap, anxiety, students were neglected, violence and discrimination against student nurses.

### Poor interpersonal relationships

During interviews participants repeatedly mentioned the issue of poor interpersonal relationships between clinical staff and students:

‘I will say the greatest experiences are that you had to deal with the attitudes of the permanent staff because a lot of times where I had moments where I just felt like quitting the course. It had something to do with the permanent staff’s attitude and the way they do things, the way they talk to you; they talk down to you like you don’t matter, you can’t even think. They down-size you so much that you don’t even feel human, you feel useless.’(Participant 29, year 4, focus group 7)

‘Actually, the staff is the main reason why I don’t want to work in a hospital. That energy that they give off is just negative. You don’t feel that you’re learning anything.’ (Participant 9, year 2, focus group 2)

### Poor communication skills

Communication between clinical staff, clinical educators and student nurses was a challenge. The following confirm ineffective communication:

‘The thing that was very negative was the staff. They think you are students you, you speak English? They aren’t really interested in you but as much as you speak Afrikaans they would say oh, and they willing to teach you everything you need to know but English – they won’t say much to you. They are not willing to show you, to teach you how things are done in a hospital setting so that’s the only negative thing to me.’ (Participant 31, year 4, focus group 7)

‘[… *S*]ome of the other staff, they don’t talk to you, they talk at you, they scream at you.’ (Participant 1, year 2, focus group 1)

‘The physios use these water bottles where the people have to blow in, but they didn’t explain to us what is that bottle for, I told the people who had the bottles that they must drink the water.’ (Participant 25, year 4, focus group 6)

### Integration of theory and practice

The nursing students are placed in healthcare settings in order to correlate theory and practice. Whilst in clinical practice, students were confused because what they learn in the classroom was not being practiced in clinical settings. The ward staff also contradict each other when doing some procedures in the clinical area, affecting the learning of students negatively.

This is what the participants had to say in this regard:

‘Sister shows me, for example how to put in a nasogastric tube. During the demonstration they told us to wear sterile gloves, then when the Sister does it, they use normal gloves, and the whole procedure for that is not aseptic. Then you feel this is not the right way, but this is how the Sister does it, then you don’t want to speak up or tell the Sister they are doing it the wrong way.’ (Participant 27, year 3, focus group 6)

‘Sometimes in certain procedures, one nurse will tell us something and another will tell us something else, and then we don’t know what to do because one says this and another one say this then you don’t know what to do who is right who is wrong.’(Participant 9, year 2, focus group 2)

### Anxiety

During clinical practice students went through a lot of stress and anxiety. According to the participants, the real situation is not like being in the simulation class. In the clinical setting they are dealing with real people; the patients’ lives are in their hands:

‘I was just so scared that I might kill a patient by doing something wrong; or doing something so wrong that I am asked to just stop training and leave.’ (Participant 29, year 4, focus group 7)

‘[… *Y*]es, Sister, and it’s very scary as well because we are dealing with people’s lives. Even if we give medication via a different route, even if it’s still in the body, you don’t know if it will have the same effect it has to have for the patient, so I have to say there was a lot of times I made terrible mistakes and I prayed that everybody stayed alive.’ (Participant 25, year 4, focus group 6)

‘I was a first-year, I had no clue what to do with an open wound. I opened the wound and there was maggots inside. I was screaming all the way and the Sister asked me what the racket was.’ (Participant 28, year 3, focus group 6)

### Students were neglected

Most of the students felt they were left alone whilst in clinical placement. They had to do the work all by themselves, without supervision of the ward staff and this had a negative impact on their learning. This was evident in the following statements from undergraduate student nurses:

‘We are a working force; the other day *ehm* – me and other student were working night shift and this staff nurse took off her shoes and sat by the nursing station the whole night whilst we were working. She actually told us in our face that we are there and they can rest when we are there so, we worked alone ….’(Participant 21, year 3, focus group 5)

‘The staff nurse was just like she and the Pen students (Hospital students nurses) were friends, were talking showing them the stuff they already know, even if you stand next to her she will call that Pen student to come and help her, and didn’t want us anywhere near her.’ (Participant 30, year 4, focus group 7)

‘They think you can do everything, so they will sit there and leave you on your own to do everything … do the vital signs, everything on your own, whilst they just sit there.’(Participant 36, year 2, focus group 8)

### Violence

According to undergraduate student nurses’ experiences, they were exposed to different types of violence in clinical settings. These students were sexually and mentally abused.

‘I was working in (operating) theater and the doctor threw gauze that was blood stained on my chest. She just threw it up in the air and it landed on my chest the bad thing she never even apologised.’ (Participant 9, year 2, focus group 2)

‘I was sexually harassed in my second year by a security guard of the hospital when we were alone. I don’t know if you know the Hospital H set-up, but it is the hospital and then across the road is the psychiatric wing. I was delivering the keys to the wing and I was alone with the security guard and he picked me up and pinned me against the wall and then he started feeling my body. That was also very emotional for me because I didn’t know how to handle it or who to go to.’(Participant 23, year 3, focus group 5)

### Discrimination against student nurses

Students mentioned that they were being discriminated against by hospital nurses:

‘The hospital students get the most support in the hospital if they want to do a procedure, but if our college students want to go and do a procedure, they don’t get the same support.’ (Participant 6, year 2, focus group 2)

‘There is a constant comparison between us and the Pen students (Pen students are nurses enrolled for Auxiliary nursing and enrolled nursing), which I think is ridiculous. You cannot possibly expect of us to be able to do those things, and first of all, nobody takes us by the hand, not even half the time that they work, so obviously, they will know the tricks of the trade. We are there for several days and just when you start getting comfortable with the staff in that particular ward, you have to leave already.’ (Participant 12, year 3, focus group 3)

### Inability to reach objectives

Students were unhappy that they were not assigned those activities that would enable them to fulfil their objectives. According to the participants they were not delegated the responsibilities wisely, and that hindered their learning:

‘In the hospital where I worked, whether you are first, second, third or fourth year, when you come to services you are expected to do vitals and to fix. You are not being allocated specifically to the area where you need to be to achieve those objectives.’ (Participant 29, year 4, focus group 7)

‘Most of the timewe as students are allocated to do observations. I am now a third-year [student] and when I go to the hospital now I will be expected to do observations. You will never get a chance to do medications or something which you have to do on a year of practice in the third year to do certain assessments.’ (Participant 11, year 3, focus group 3)

### Congestion of students in a clinical setting

Large numbers of students in one placement area decreases the learning opportunities for student nurses. Below are the comments of the participants:

‘I haven’t had a good experience in the hospital. It’s like we had two types of students, the Pen students (Pen students are nurses enrolled for Auxiliary nursing and enrolled nursing) and us. I was about to do a procedure on one of the patients when another student said, “No, that’s my patient.”’ (Participant 19, year 3, focus group 4)

‘In the hospital, we are up to seven students in a ward from first to the fourth year, so there is one or two Sisters in the ward, excluding the Operational Manager, of course, so it’s difficult for her to teach a first-year and a second year at the same time, because it’s two different things.’ (Participant 1, year 2, focus group 1)

## Discussion

The aim of this study was to explore and describe the experiences of the undergraduate nursing students during clinical practice in Boland Overberg area, in Western Cape. The results of this study revealed that students experienced different challenges whilst in various clinical settings. According to the study participants, these challenges led to a non-conducive learning environment, which hindered the student learning. These findings support those of Jamshid’s (2016:2), who stated that challenges in clinical settings can prevent student nurses from effective learning and growth. It was found that the challenges encountered by student nurses, affected the student nurses negatively and hindered their learning.

### Interpersonal relationships

Most of the students experienced inappropriate, interpersonal relationships with clinical staff. The findings of the current study revealed that, the ward staff were disrespectful and mean to the student nurses. This negative attitude of ward staff towards nursing students undermined the student nurses’ trust and respect for professional nurses. These findings concur with those of Matshotyana et al. (2015:118); Motsilanyane (2015:83); Mamaghani et al. (2018:219) and Moghaddam et al. (2020:316), who stated that relationships between nursing students and ward staff were poor. Participants of the current study reported that clinical staff was unapproachable and shouted at the students when they did something wrong, not listening to the explanations given by the students.

The current study revealed that the staff’s attitudes and perceptions had a negative impact on the learning of these nursing students. Both parties, student nurses and clinical staff, failed to set goals and make decisions together. Students were ignored by the clinic staff, even when they asked for help and were instead ridiculed by them.

These findings are consistent with those of Lee, Clarke and Carson (2018:107), who reported that poor interpersonal relationships between clinical staff and student nurses in placement areas hinder the learning process of student nurses. It also does not set a good example to these students in forming good relationships and developing interpersonal skills. During the interviews, some participants reported that they wanted to quit nursing because of the negative attitudes of the staff towards them. The current study is consistent with that of Kalyani et al. (2019:2), who reported that relationships with clinical staff were a major challenge affecting students’ learning and interpersonal relationships. This study revealed that the students were discouraged from asking questions when they faced problems during clinical practice in the wards because of the negative attitudes of clinical staff. An unsupportive negative attitude results in the deterioration of the educational quality in the clinical environment, and this discourages nursing students (Matshotyana et al. 2015:118; Motsilanyane 2015:84).

### Poor communication

Communication is the cornerstone of nursing services (Jooste 2018:230). In the current study, student nurses found that communication was ineffective. This brought frustration and stress amongst student nurses and clinical staff. Because of poor communication, staff members were not prepared to exchange information or guide the student nurses (Mamaghani et al. 2018:219). Poor interpersonal communication skills can affect people’s social exchanges, as was the case in this study, where students were too scared to communicate with ward staff, or ask any questions (Mamaghani et al. 2018:219). One of the characteristics of effective communication is listening. The study revealed that clinical staff ignored the student nurses, even when these nurses gave feedback about patient care that had a bearing on the patient’s health. Good communication improves the quality of care provided to patients (Mamaghani et al. 2018:219). A study done in Iran, Moghaddam et al. (2020:316), reported that ineffective communication between clinical staff and student nurses is a barrier to learning and it breaks the trust between clinical staff and a student nurse.

According to Jooste (2018:229), a lack of communication creates a situation where medical hazards can occur. Because of poor communication, the patient’s life could be compromised. The clinical learning opportunities, clinical guidance and the support mechanisms in place for these nursing students are hampered by ineffective communication (Jahanpour et al. 2016:2).

According to Mikkonen et al. (2016:181), differences in language and culture in healthcare facilities impede the learning of student nurses. The participants reported having missed learning opportunities in the wards owing to language problems. It was found that the clinical staff avoided nursing students who could not express themselves in Afrikaans. Some of the participants reported that ward staff focused only on those students who were able to speak and understand Afrikaans. According to Furnes, Kvaal and Høye (2018:1), nursing students speaking a vernacular language, other than that of the majority of their peers, might face an additional challenge when it comes to communication skills. Not being proficient in the commonly spoken language made the students feel frustrated and isolated. Language problems can be a barrier to quality patient care. Students reported that they had a problem reading documentation, and during handover and ward rounds, they missed important information as they did not understand the language used by the staff.

### Integration of theory and practice

The literature revealed a gap in integrating theory with practice. This has been a problem for a long time in nursing education (Kaphagawani & Useh 2018:108; Mothobi 2017:80; Tang & Chan 2019:6; Zenani 2016:43). According to the participants in this study, there were discrepancies between what they were taught and what was practiced in the clinical settings. This theory-practice gap hindered the student clinical learning.

These findings support those of Jahanpour et al. (2016:2), who reported that students in Iran complained of theory gaps in practice, which left them confused about how to do the procedures. Participants in this study were disappointed when they arrived at the clinical settings because they expected to put into practice what they had learned in the classroom. They found the procedures were done differently from what they had learned in the classroom and the simulation laboratories. Similar results were found by Kaphagawani (2015:152) in Malawi. This author reported that when students reached the clinical settings, procedures were done differently from what they had learned at college. This study revealed that there were conflicting practices amongst clinical staff. One sister would tell the student something, and the other would say something different, resulting in confusion and frustration amongst the students. These findings concur with those of Kaphagawani (2015:152) that conflicting practices between the ideal nursing taught in classrooms and that being practiced in clinical settings, result in students being confused and anxious.

Participants in this study reported that clinical staff were task-oriented and did not care for patients in accordance with their needs (as they had been taught before being placed in these clinical settings). These findings were similar to those of Zenani (2016:47), where the participants reported that the care provided to patients by clinical staff, was task-orientated and did not resemble the comprehensively taught skills and embedded knowledge obtained before they entered the clinical setting.

### Anxiety

Sun et al. (2016:22) describe anxiety as a feeling of fear, uneasiness and uncertainty. According to these authors, anxiety is usually accompanied by negative emotions such as feeling uncomfortable or unhappy (Sun et al. 2016:22).

This study revealed that clinical practice was stressful. Some of the participants were anxious because they were dealing with patients’ lives. They feared something might go wrong, resulting in the death of the patient. These findings correspond with those of Sun et al. (2016:25), which showed that student nurses in their clinical practice were worried about causing harm to the patients. The participants reported that they felt anxious because they were unsure of whether they would be able to deliver what was expected of them. These findings are consistent with those of Dinmohammadi, Jalali and Peyrovi (2016:34), who reported that students were worried about being faced with patients’ needs they could not deal with and feared making mistakes that could harm patients. The current study revealed that the real-life situations they were in caused anxiety, as opposed to handling mannequins in simulation laboratories. This study supports that of Ahmadi et al. (2018:68), who found that the reality of practice could cause anxiety for students and hinder the implementation of skills learnt in practice.

### Students were neglected

The participants in this study reported that students were left alone to do work without supervision. This put a lot of pressure on students because they were frightened of making errors. The literature reveals that on many occasions, students are left alone to struggle with unfamiliar tasks (Moghaddam et al. 2020:316). Atakro et al. (2019:7), concur with these findings, stating that leaving students unattended with patients can put the patients’ lives at risk. Some literature reported that if students are left alone in placement areas with little support from clinical staff, it impacts negatively on their confidence (Kaphagawani & Useh 2018:106). This study revealed that because these students were ignored, they also lost confidence in nursing. Some of the participants reported that clinical staff wanted to have nothing to do with them. Atakro et al. (2019:7), concur with these findings, stating that clinic staff had no interest in the students and that made students feel that they were a burden to the ward staff. Busy wards were one of the factors identified as the cause of students being left alone (Neshuku & Amukugo 2015:89). Atakro et al. (2019:6), in their study, revealed that the generation gap between the undergraduate nursing students and clinical staff also lead to the isolation of student nurses. This generation gap brought clashes between the different generations, as these student nurses come with new ideas in the clinical areas (Atakro et al. 2019:6).

### Violence

Tee, Özçetin and Russell-Westhead (2016:30), define workplace violence as a violent act directed towards workers. It can include physical, psychological or verbal behaviour. According to Webster et al. (2016:40), violence represents negative interactions involving any form of bullying, abuse, harassment or unwanted behaviour that can cause the recipient to feel unwanted, threatened or upset. Tee et al. (2016:30) state that violence can include physical, psychological or verbal behaviour. Participants in this study experienced different types of abuse. Some reported that they were sexually abused by support staff which included security, clerks and other administrative staff. The findings of this study are similar to those of Engelbrecht, Heyns and Coetzee (2017:8494) who reported that most undergraduate nursing students in South Africa experience intra-professional violence in the clinical environment. Findings of this study revealed that students were also verbally and emotionally abused by the clinical staff and the patients placed in their care. Most of these participants were humiliated, belittled and talked about in a derogatory manner behind their backs. These findings are consistent with those of Webster et al. (2016:44) where students were bullied, yelled at, belittled and had sarcastic remarks made to them. Participants in this study reported that clinical staff was hostile, rude and verbally abusive. These findings are similar to those of Vuolo (2018:107) in the United Kingdom, where students reported being vulnerable, as they were bullied and belittled by clinical staff. According to Mamaghani et al. (2018:221), similar negative experiences amongst students can have a direct impact on the development of their future professional skills and competencies as nurse practitioners.

### Discrimination against students

Discrimination against nursing students in clinical settings is one of the challenges that nursing students encountered during their placements. This discrimination occurs in healthcare systems and unfortunately, nursing staff are involved in exacerbating this discrimination against nursing students (Mamaghani et al. 2018:220). This study revealed that students were being discriminated against because of the college where they were undergoing their nurse training. These findings were consistent with those of Engelbrecht et al. (2017:8494), who reported that students were discriminated against based on their place of training. Staff at the institutions preferred supervising their own students training at their hospital nursing schools. These include lower categories of nursing courses, compared with the participants enrolled for a degree course, who needed more sophisticated and in-depth supervision. According to Jamshidi et al. (2016:4) and Mamaghani et al. (2018:220), nursing students are sometimes compared unfavourably with students in other fields who are also placed in the same settings, for example medical students, physiotherapists and occupational therapists. Participants in this study reported that they were refused the use of equipment like the Dinamap, whilst hospital students could use the equipment. These practices disadvantaged students from the college tremendously, as they could not experience utilising important equipment as part of rendering quality nursing care. Mamaghani et al. (2018:220) in their study found that student nurses had experienced discrimination in respect to the distribution of resources as well, as was the case in this study. Participants of this study reported that they were treated unfairly by clinical staff: other students studying other nursing programmes received better attention in terms of support and supervision.

### Inability to achieve objectives

The *Nursing Act* No. 33 of 2005, states that students should acquire the learning outcomes set in a specific, identified field as part of their training, in order to be regarded competent to practice as professional nurses. This study revealed that students were unable to achieve their clinical objectives. According to the findings of this study, an inappropriate delegation of students had a negative impact on the students’ learning outcomes. Since the objectives were not considered prior to their delegation the students were not assigned to their stations according to the set clinical learning objectives of their curriculum. Kaphagawani (2015:211) reported that students were expected to be placed in work areas so that they would accomplish their learning objectives. When students were in clinical areas, they were expected to do routine jobs, even if those jobs were not on their learning objectives. The participants of this study reported that sometimes they were removed from their placement areas and that made it difficult for them to achieve their learning objectives. Kaphagawani (2015:211) highlighted the importance of allocating student nurses in clinical areas aligned to their learning objectives in order to practice what they had learned in class for optimal gain in knowledge. As a result of excessive workloads and staff shortages, students were forced to perform duties beyond their scope of practice and this hindered the students from accomplishing their learning outcomes (Letswalo & Peu 2015:360). Clinical units should not forget that students are placed and rotated regularly amongst the various areas in order to achieve their objectives. Students should not be removed from where they are allocated to work for a certain period, and they should not be used to replace permanent staff in another department. It is important to expose these students to quality care in order to produce competent nurses that we will be proud of. To ensure that students achieve their objectives, nurse educators should be available whilst students are delegated to clinical settings and they should support the student nurses (Muthathi, Thurling & Armstrong 2017:e6.).

### Congestion of students

Participants in this study reported that large numbers of students in one placement area limited their learning opportunities. Similar findings were also found in the study of Ahmadi et al. (2018:66), where participants reported that they had problems in accessing clinical learning opportunities owing to the high numbers of students in the clinical practice areas. The findings of this study revealed that because of the high number of students in one clinical area, students had to compete for limited procedures. Kaphagawani and Useh (2018:108) concur with the aforementioned findings as they reported that there were too many students on the ward competing to do procedures, which hindered the student nurses’ learning. The literature revealed that large numbers of students in clinical settings affect the learning of student nurses negatively (Ali & Ali 2017:75; Kaphagawani 2015:224). Participants in this study reported that congestion of students made it difficult for the registered nurses and mentors to teach and supervise them appropriately. These findings are consistent with those of Luhanga (2017:98), who noted that too many students make supervision difficult and increase the risk of errors. According to Troung (2015:90), in those wards with small numbers of students, preceptors had more opportunity to provide students’ learning needs compared to those clinical areas that often had large numbers of students. Houghton et al. (2013:1966) in their study reported that students tend to isolate themselves if placed in a single clinical setting in large numbers, and they ultimately withdraw from potential supportive relationships in such situations. This study revealed that if there are large numbers of students in one ward, it does not matter whether those students are from different programmes; the students get confused and don’t know what to do. According to the participants in this study, because of the large number of students allocated to one ward, clinical staff relaxed and students had to do all the work alone without direct supervision.

## Conclusion

The current study revealed that undergraduate students had challenging experiences during clinical practice in the healthcare settings. The outstanding challenges were integration of theory and practice, anxiety, students neglect, violence, congestion of students and discrimination of students; interpersonal relationships, poor communication and inability to reach objectives. These challenges affected learning of undergraduate student nurses negatively. The participants of this study believe that change of clinical staff attitudes and reduction of the gap between theory and practice can lead to a conducive learning environment.

### Implications

Findings of this study can be used in planning of nursing education clinical curricula; could provide help to develop effective clinical teaching strategies in nursing education as well as to support these undergraduate nursing students.

### Limitations

One nursing school was included in this study, hence no generalisation.

### Strengths

Recruitment process and data collection were made easy by the fact that students who were the participants of this study, were eager to take part in the study.

### Recommendation

All categories of staff involved with the experiential training of students need regular updates on all nursing procedures. This includes lecturing staff, so that all persons involved are aware of the correct way in which nursing care should be rendered and how procedures should be managed and executed. This will help to bridge the gap between theory and clinical practice.

Regular interpersonal skills training for staff should be arranged, in order to reduce poor attitudes and behaviour towards students in training.

Large numbers of students in one unit make it difficult for the clinical staff to supervise students. Therefore, when placing students, the number of students should be controlled.

Effective communication skills can be taught when nursing students are doing interpersonal skills, before they go to the clinical environment. Before the nursing students go to the clinical environment, the difference between the clinical environment and classroom environment must be emphasised. Before the nursing students go for their clinical practice to the real environment, they must get psychological counselling and visit the clinical environment prior to their placement, in order to familiarise themselves.
